# Cannabis use, health problems, and criminal offences in Germany: national and state-level trends between 2009 and 2021

**DOI:** 10.1007/s00406-024-01778-z

**Published:** 2024-03-19

**Authors:** Jakob Manthey, Sinja Klinger, Moritz Rosenkranz, Larissa Schwarzkopf

**Affiliations:** 1 Centre of Interdisciplinary Addiction Research (ZIS), Department of Psychiatry and Psychotherapy, University Medical Center Hamburg-Eppendorf (UKE), Martinistraße 52, 20246 Hamburg, Germany; 2 Institute for Interdisciplinary Addiction and Drug Research, Lokstedter Weg 24, 20251 Hamburg, Germany; 3 Department of Psychiatry, Medical Faculty, University of Leipzig, Semmelweisstraße 10, 04103 Leipzig, Germany; 4 IFT Institut Für Therapieforschung, Mental Health and Addiction Research, Leopoldstrasse 175, 80804 Munich, Germany; 5 Department of Psychiatry and Psychotherapy, University Clinic of the Ludwig Maximilians University Munich, Ziemssenstrasse 5, 80336 Munich, Germany

**Keywords:** Cannabis, Legalisation, Criminal justice, Treatment demand, Prevalence

## Abstract

The German federal government plans to decriminalise cannabis. The impact of this policy on use prevalence, cannabis-related health and legal problems cannot be fully anticipated and should be viewed in context with current trends. We used routine data on (a) cannabis use (population-based surveys), (b) cannabis-related diagnoses (ICD-10 code F12) in outpatient medical settings and (c) minor law offences (registered violations against the narcotics law for possessing small amounts) to analyse age and sex-specific trends by federal state between 2009 and 2021. To enable comparisons across time and federal state besides crude prevalence rates, age-standardised rates were calculated. Between 2009 and 2021, the age-standardised prevalence of cannabis use (5.7–10.6%), rate of diagnoses (1.1–3.7 per 1,000), and legal offences (1.8–3.1 per 1,000) increased, with the largest increase noted for cannabis-related diagnoses. Relatively, increases were most pronounced for older users (40-to-59-year-olds: use and offences; 35-to-44-year-olds: cannabis-related diagnoses) and rather stagnant for minors. Cannabis use and health problems appear to be more pronounced in Northern and city states, while no clear geographic trend was observed for law offences. Cannabis-related outpatient treatment demand has risen more steeply than use prevalence suggesting an increasing challenge for the health care system. Despite rising rates for documented offences, the long-term implications of law violations on social and occupational life are poorly understood but may be considered for evaluations of the proposed law changes.

**Supplementary Information:**

The online version contains supplementary material available at 10.1007/s00406-024-01778-z.

## Introduction

Cannabis is by far the most prevalent illegal drug in Europe and in most countries globally with an estimated 220 million people worldwide having used cannabis in 2021 [43], with about 23 million people who use cannabis living in Europe [11]. Here, the past-year prevalence of use has increased from about 5.6% to 6.8% between 2010 and 2019 among 15- to 64-year olds [22].

Several social and health risks are associated with cannabis use [6]. Early-onset cannabis use increases the risk of dropping out of school and of attaining lower levels of education [42]. Among adults, regular cannabis use has been linked to decreased employment chances [10]. Health risks related to cannabis use concern mostly mental health outcomes for which the risk increases with intensity of use (for cannabis use disorder/CUD: [20]; for psychosis: [38]). More recently, a connection between cannabis with suicide [7, 39] and cardiovascular health [44] was stated.

In addition to social and health consequences in context of cannabis use, further risk arises from the legal framework. Most importantly, the full-scale prohibition has facilitated the emergence of synthetic cannabinoids—mimicking the effects of natural cannabinoids—which are apparently more harmful than natural cannabinoids [2]. Moreover, people using cannabis are at risk for being arrested for possession of minor amounts, depending on the laws in their country. While most drug-related offences in Europe will not result in imprisonment, there might still be grave social consequences (e.g. job loss).

According to long-term follow-ups following the commercial legalisation of cannabis markets in several US states and Canada, cannabis use has increased among adults and adolescents and there are additionally indications for increased cannabis-related hospitalisations [23]. Setting out to learn from mistakes in other countries, Germany pursues a middle-ground option between prohibition and commercialisation: following a liberalisation of the medical market in 2017, the federal government plans to decriminalise the possession of small amounts of cannabis for recreational purposes. Moreover, the current plans allow the cultivation of a limited number of plants for adults or for non-profit cooperatives. Finally, pilot studies to evaluate the legal, commercial sale of cannabis in geographically limited regions are considered [4].

To evaluate the proposed law changes, a robust understanding of historical or secular trends is required. As Germany is the most populous country in Europe and has considerable regional differences, rooted in the historical divide of the country but also in the federal system, granular data beyond the national level appear warranted. In this contribution, we assess (1) cannabis use prevalence, (2) cannabis-related diagnoses, and (3) offences for possessing cannabis stratified by sex, age, and federal state to examine trends 2009 and 2021.

## Methods

### Cannabis use prevalence

Two population-based, nationwide surveys have collected self-reported information on substance use in the past decade. The first is conducted triennially, focusses on 18- to 64-year-olds (estimates for 2021: [34]; trends up to 2018: [41]), and provides estimates for the entire country and for select states in some years. The second survey is held bi- or triennially and focuses on 12- to 25-year-olds [29].

We combined the data of both surveys to estimate the annual 12-month cannabis use prevalence for each federal state, by sex and 4 age groups (12–17, 18–24, 25–39, 40–59) for all years 2009 to 2021. For several states, prevalence data were lacking. To close this gap, we assumed geographical variation in cannabis use to be proportional to the geographical variation in hospitalisations due to CUD (ICD-10 diagnosis code F12; see Supplementary Fig. 1 for an illustration of the trends in hospitalisation rates for each federal state). This assumption held true for states with observed data and was extrapolated to those states with missing data. More information on the estimation strategy is provided in Supplementary Information and Supplementary Figs. 2, 3, 4, 5.

### Cannabis-related legal problems

The number of people who have violated the narcotics law in Germany were taken from the annual police crime statistics which discloses the number of offences, stratified by type of offence, state, sex, and age groups [3]. We restricted the analyses to offences related to possessing minor amounts of cannabis for personal consumption (223,226 persons in 2021; 69% of all cannabis-related offences). Thus, we excluded offences that involved any illegal production of, trade with, or smuggling of cannabis.

### Cannabis-related diagnoses

Cannabis-related diagnoses were defined as outpatient ICD-10 diagnoses of CUD (‘*mental and behavioural disorders due to use of cannabinoids’)*. We obtained the number of people insured with the statutory health insurance (covering about 85% of German residents [5]) that received an F12 diagnosis in medical outpatient settings (general practitioners, medical specialists, psychotherapists) at least once per calendar year, by state, sex, and age group (15–19, 20–24, 25–29, 30–34, 35–44, 45 +). In Germany, the rate of F12 hospitalisations for people aged 65 or older approaches 0 (compared with 45-to-64-year-olds: 4; [16]), thus, we assumed that all people with F12 diagnoses in age group 45 + were at most 64 years old. Across all years, 3 out of 10 diagnosed persons were recognised by psychotherapists, while the remaining cases were diagnosed by physicians of different professions. For each calendar year, the number of people with a respective diagnosis was identified, but it should be noted that some people were diagnosed in more than 1 year (total number of unique persons identified across all years: 559,174; sum of annual figures: 1,333,842). The data on F12 diagnoses were provided by the Central Institute of the Federal Association of SHI physicians and rely entirely on confirmed or suspected diagnoses.

Data from inpatient settings were deliberatively disregarded for two reasons: first, cannabis-related health problems comprise mostly mental health problems that are rarely linked to somatic comorbidities which would warrant inpatient treatment. Second, we employed inpatient data for imputing use prevalence estimates for states with missing data. Including hospitalisation data as additional indicator, we would expect the prevalence of use and hospitalisation to be associated as they are inherently linked with each other, limiting our interpretation of three independent data sources. Nevertheless, the hospitalisation data are presented in Supplementary Fig. 1 for context.

### Statistical analysis

For use prevalence and offences, we used the same age groups (12–17, 18–24, 25–39, 40–59) but for cannabis-related health problems, we applied—owing to data availability—a slightly different grouping (15–19, 20–24, 25–29, 30–34, 35–44, 45–64). For age-specific estimates, we report crude rates (% for prevalence, per 1,000 for diagnoses and offences). For estimates across all (available) age groups, we report age-standardised rates which remove the (possible) impact of age variations and thus facilitate comparisons across time and federal states.

To calculate age-standardised rates, weights were determined by age distribution in Germany observed in the year 2021: for the prevalence of cannabis use and the rates of offences for cannabis possession, the German resident population served as resident population. For cannabis-related diagnoses, the reference population was the SHI-insured population obtained from the federal ministry of health [5].

We performed mostly descriptive analyses in addition to some correlations. All analyses were conducted in R version 4.2.3 [33] and the input data as well as the R code used to produce the findings and graphs are publicly available (https://www.doi.org/10.6084/m9.figshare.23850840).

## Results

### National distribution

In 2021, the (imputed) age-standardised 12-months cannabis use prevalence among 12–59-year-olds was estimated at 10.6%, which translates into an estimated 5.2 million people who use cannabis (2009: 2.9 million). At the same time, about 170,000 SHI-insured people received at least one F12 diagnosis in outpatient settings (age-standardised rate of cannabis-related diagnoses: 3.7 per 1,000) and 152,000 cannabis-related offences were registered by the police (age-standardised offence rate: 3.1 per 1,000).

**Fig. 1:**
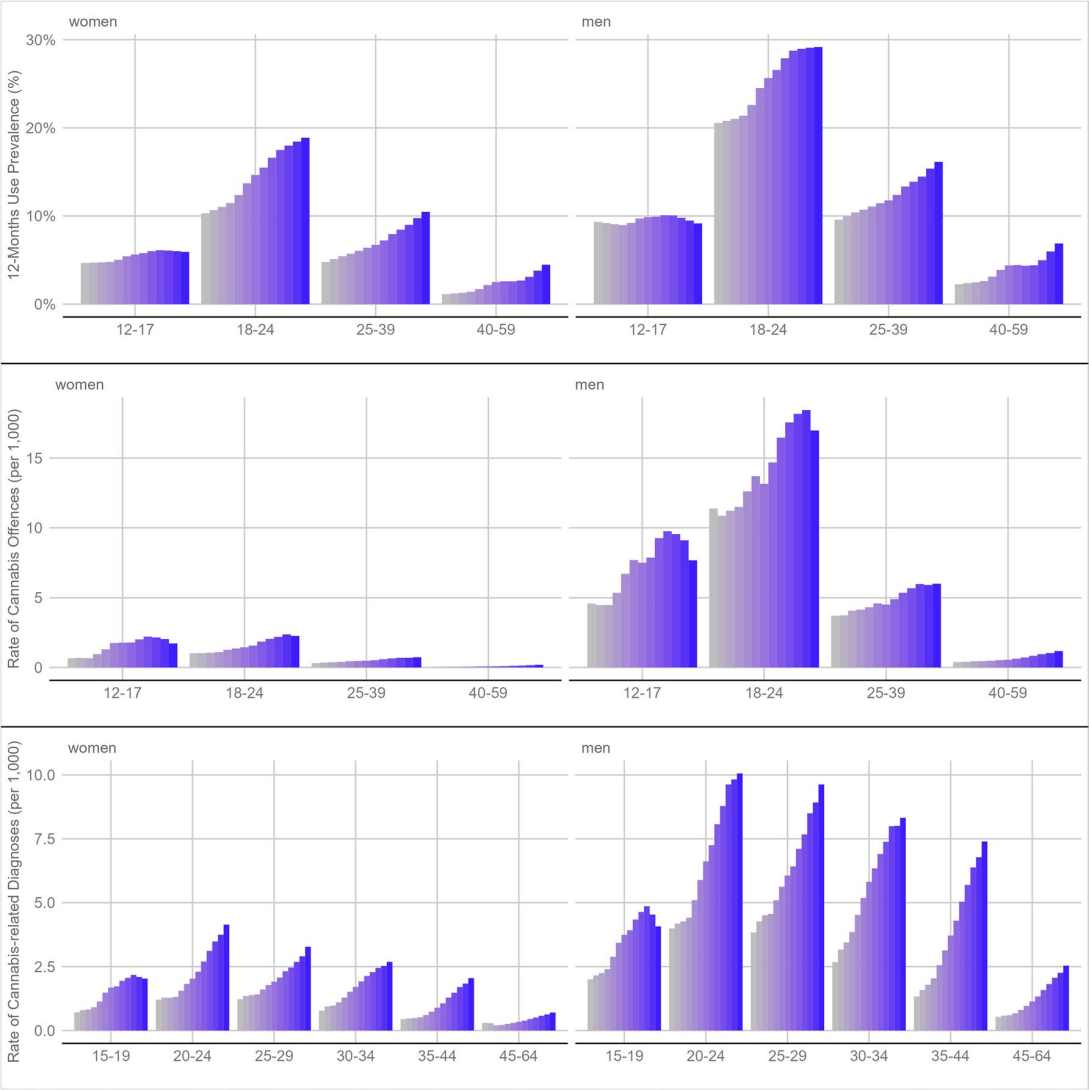
Cannabis use prevalence (top; imputed data), cannabis-related offences (middle; registered data), and cannabis-related diagnoses (bottom; registered) by sex (women: left; men: right) and age group (x axis). Each bar represents 1 year (start: grey in 2009; end: blue in 2021). Note that the y-axis in each plot varies by row to accommodate variables with various scales

A break-up of the data at the national level by year, sex, and age highlights two patterns (see Fig. 1). First, cannabis use, cannabis-related diagnoses and cannabis-related offences were most prevalent among younger adults. However, the two youngest age groups made up only about one-third of the users in 2021 (12–17 years: 6% of all users; 18–24 years: 28% of all users). Most cannabis users were 25–39 years of age (40% of all users), every fourth user belonged to the oldest age group (40–59 years: 25% of all users). Comparing the age structure of users and offenders, younger age groups are overrepresented among offenders (see Supplementary Fig. 6). Moreover, in 2021, the male–female-ratio was highest for the offence rate (7.1:1), followed by the rate of cannabis-related diagnoses (3.2:1), and lowest for use prevalence (1.6:1; for an age break-up of the sex ratio, see Supplementary Fig. 7). In other words, cannabis users are most likely to be arrested or fined for violating the narcotics law if they are young and of male sex.

Second, between 2009 and 2021 all three indicators exhibit increases of varying magnitude across sex and age groups (see also Table 1): the age-standardised prevalence of cannabis use (from 5.7% to 10.6%) and the rate of cannabis offences has nearly doubled (from 1.8 to 3.1), while the age-standardised rate of cannabis-related diagnoses has nearly tripled (from 1.1 to 3.7 per 1000 insured population). The rises in all three indicators were primarily driven by older age groups. On the contrary, cannabis use, offences, and cannabis-related diagnoses among minors have stayed stable or even decreased in the most recent years.

**Table 1:** Age-specific point estimates of cannabis use prevalence (imputed), F12 diagnoses (registered), and cannabis-related offences (registered) in 2009 and 2021

Indicator	Age	Absolute numbers			Prevalence/rate per 1000^1^		
		2009	2021	Relative change	2009	2021	Relative change
12-month cannabis use	12–17	342,893	342,398	0%	7.1%	7.6%	+ 8%
	18–24	1,055,718	1,476,766	+ 40%	15.5%	24.2%	+ 56%
	25–39	1,079,157	2,112,648	+ 96%	7.2%	13.4%	+ 86%
	40–59	430,539	1,309,831	+ 204%	1.7%	5.7%	+ 235%
	*12–59*^*2*^	2,908,307	5,241,643	+ 80%	5.7%	10.6%	+ 88%
Offences for possessing cannabis	12–17	13,003	21,594	+ 66%	2.7	4.8	+ 79%
	18–24	42,914	60,525	+ 41%	6.3	9.9	+ 57%
	25–39	30,445	54,414	+ 79%	2.0	3.4	+ 69%
	40–59	5,746	15,768	+ 174%	0.2	0.7	+ 202%
	*12–59*^*2*^	92,108	152,301	+ 65%	1.8	3.1	+ 73%
Cannabis-related diagnoses	15–19	5,220	10,220	+ 96%	1.4	3.1	+ 125%
	20–24	11,039	28,453	+ 158%	2.6	7.2	+ 178%
	25–29	10,649	28,453	+ 167%	2.5	6.6	+ 162%
	30–34	6,732	27,850	+ 314%	1.7	5.6	+ 228%
	35–44	8,587	44,269	+ 416%	0.9	4.7	+ 443%
	45–64	7,772	32,998	+ 325%	0.4	1.6	+ 288%
	*15–64*^*2*^	49,999	172,243	+ 245%	1.1	3.7	+ 239%

^1^Prevalence for 12-month cannabis use and rate for cannabis offences and cannabis-related diagnoses

^2^Prevalence/rate estimates are age-standardised for age groups covering all ages (use and offences: 12–59; cannabis-related diagnoses: 15–64)

### Regional distribution

**Fig. 2:**
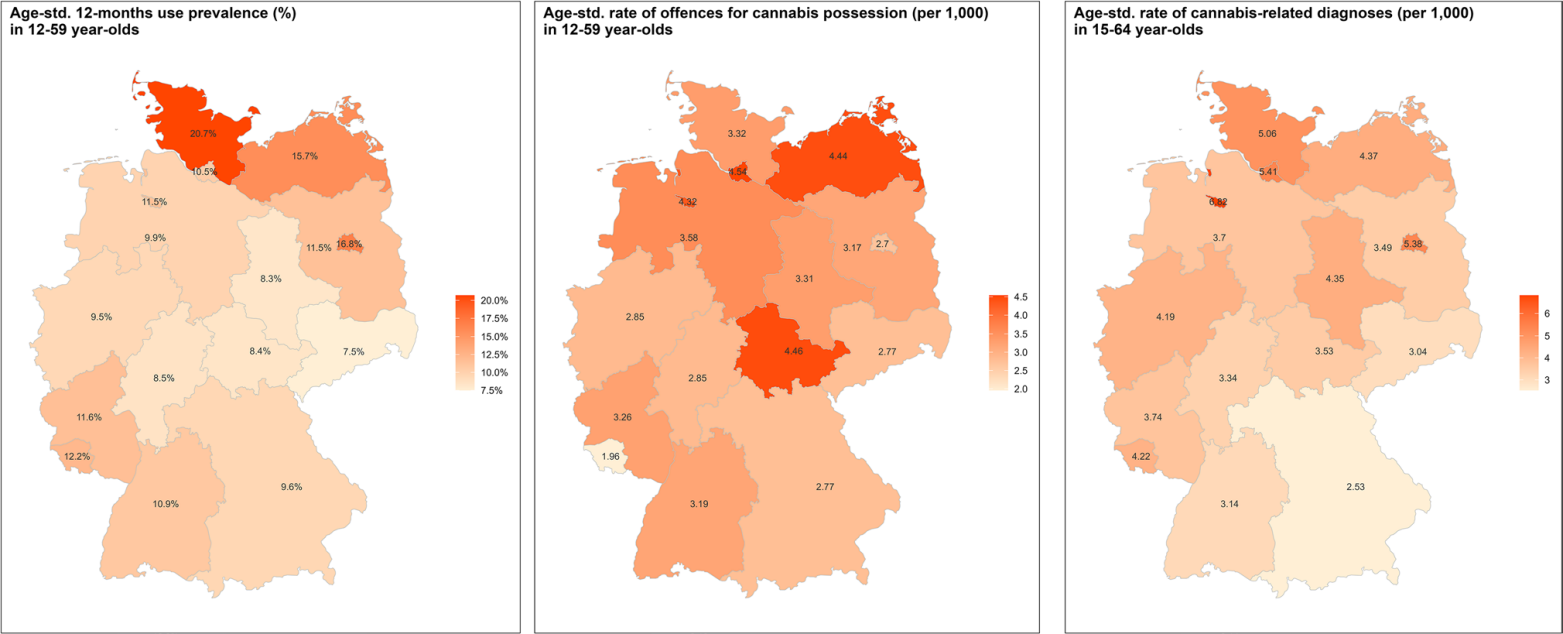
**a** Age-standardised 12-month prevalence of cannabis use (left; imputed), **b** rate of offences for possessing cannabis (middle; registered), and **c** rate of cannabis-related diagnoses in outpatient settings (right; registered) in 2021, by federal state

As shown in Fig. 2, the prevalence of use and rates of cannabis-related diagnoses were higher in Northern states and in the three city states, as compared to Eastern and Southern regions. The age-standardised cannabis use prevalence was highest in two Northern states (Schleswig–Holstein: 20.7%; Mecklenburg-Western Pomerania: 15.7%) and the capital Berlin (16.8%) and lowest in three East German states (Saxony: 7.5%; Saxony-Anhalt: 8.3%; Thuringia: 8.4%). For the administrative, age-standardised rate of outpatient cannabis-related diagnoses, the highest rates were observed in the three city states (Bremen: 6.9 per 1000; Hamburg: 5.4; Berlin: 5.4), and the lowest rates in the two Southern (Baden-Württemberg: 3.1 per 1000; Bavaria: 2.5) and one Eastern state (Saxony: 3.0). For offences, there was no clear geographical gradient: two city states had one of the highest (Hamburg: 4.5) and lowest (Berlin: 2.7) offence rates as did two neighbouring East German states (Thuringia: 4.5; Saxony: 2.8). The lowest offence rates were observed in a southwestern state (Saarland: 2.0).

For cannabis use and cannabis-related diagnoses, there appears to be a moderate spatial correlation (Pearson r = 0.47), suggesting above-average use and diagnosis rates in Northern and city states and below-average use and diagnosis rates in Southern/Eastern states. Also, a moderate spatial correlation (Pearson r = 0.40) was observed for offence and diagnosis rates. In contrast, cannabis use prevalence and offences did not correlate at all (Pearson r = 0.03).

**Fig. 3:**
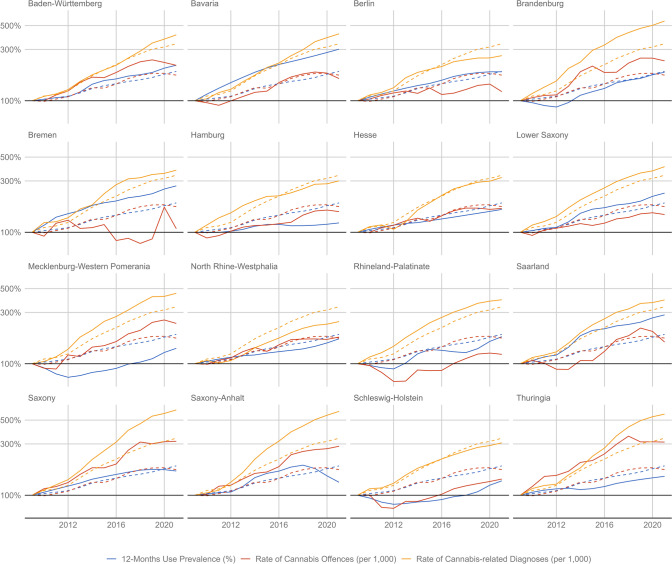
Change in the age-standardised **a** 12-month prevalence of cannabis use (blue; imputed), **b** rate of offences for possessing cannabis (red; registered), and **c** rate of cannabis-related diagnoses (orange; registered) between 2009 and 2021. The dashed line indicates the national average for each indicator. For each state and indicator, the data are normalised to 100 for the year 2009, i.e. 300% indicates a tripling of the value relative to the year 2009. Note that the *y*-axis is logarithmised for improved illustration

As shown in Fig. 3, the magnitude of rising cannabis indicators varies substantially across the 16 federal states. For the age-standardised 12-month cannabis use prevalence, we observed little changes in Hamburg (+ 22%) and Saxony-Anhalt (+ 32%), while Bavaria (+ 201%) and Saarland (+ 184%) saw more substantial increases (national average: + 87%). For the age-standardised rate of offences for possessing cannabis, Bremen (+ 8%) and Berlin (+ 21%) showed little change, while substantial increases above the national average (+ 73%) were observed in four East German states (Mecklenburg-Western Pomerania: + 137%; Saxony-Anhalt: + 186%; Thuringia: + 212%; Saxony: + 218%). For the age-standardised rate of cannabis-related diagnoses in outpatient settings, below-average (national average: + 239%) increases were found in the most populous state North Rhine-Westphalia (+ 147%) and in the two cities Berlin (+ 163%) and Hamburg (+ 201%). In contrast, the three East German states with the lowest use prevalence in 2021 saw the largest increases: Saxony (+ 522%), Saxony-Anhalt (+ 503%), and Thuringia (+ 468%).

## Discussion

In this study, we delineate trends of cannabis use, offences for possessing minor amounts of cannabis, as well as cannabis-related diagnoses in Germany. Between 2009 and 2021, the estimated number of people using cannabis has increased from about 2.9 to 5.2 million while the number of people with a cannabis-related diagnosis in outpatient medical settings has more than tripled, thus, exceeding the growth of users. Out of the three indicators examined, offences for possessing cannabis have increased the least. Hence, the proportion of cannabis users seeking treatment has increased while the proportion of cannabis users registered to have violated the narcotics law has decreased slightly.

### Limitations

Before discussing the implications of these trends and the subnational differences, we want to highlight some limitations. First, the estimates of past-year use prevalence are obtained from two population-based surveys which may considerably underestimate the true prevalence of cannabis consumption [1]. While population-based surveys do constitute the gold standard in the field, the validity of their estimates is determined by sampling frame and response biases. However, using data from the same surveys with a comparable methodology over time and assuming that the biases do not change substantially over a period of 10 years, we presume that the time trends of our estimates are consistent. Second, for those states without any observed prevalence rates, the estimates rely strongly on the assumption that use prevalence and cannabis-related hospitalisations correlate substantially. Unless any surveys are conducted in those states, we are unable to validate our findings, thus, they should be interpreted with caution. For states with available survey estimates, the imputed values appear to be generally close to the observed data (see Supplementary Fig. 1) thus our assumption of correlating use prevalence and CUD-related hospitalisation appears dependable from a methodological point of view. Third, the rising rate of cannabis-related diagnoses may reflect greater awareness of physicians and psychotherapists or an increased tendency to seeking help on the users’ side. With the data available, we are unable to disentangle these two drivers. Fourth, we do not have detailed information on the extent of synthetic cannabinoid use in Germany, so we are unable to accurately describe the share of cannabis-related diagnoses that reflect chronic use of natural vs. synthetic cannabis products. In the 2017/2018 European Web Survey of Drugs, there were 27,231 persons reporting use of only natural cannabis products contrasting 104 persons who reported using only synthetic cannabinoids (2,567 used both: [21]). As a sizeable share of European cannabis samples suspected for adulteration do contain synthetic cannabinoids [28], the number of people who unwillingly use synthetic cannabinoids may be higher than reported by users. In German specialised outpatient addiction care facilities (not part of the analysed data as they are not funded by SHI), only 0.1% of admissions declare “synthetic cannabinoids/other” as main drug in 2021 (compared with 23.3% cannabis: [40]). Also, UK hospital data show no notable upward or downward trend between 2015 and 2019 [9]. Acknowledging that the acute use of synthetic cannabinoids is associated with greater adverse somatic consequences than the acute use of natural cannabinoids [8], we believe that the contribution of synthetic cannabinoids to total cannabis consumption and resulting chronic health problems (cannabis-related diagnoses in outpatient settings) is minor.

### Implications

The trends of all three indicators suggest rising cannabis use as well as increasing cannabis-related legal and health problems. Trends regarding rising cannabis use [29, 41], cannabis-related hospitalisations [15], and treatment demand in specialised addiction centres [22] have been described before. Our study adds that the number of persons with cannabis-related diagnoses by physicians and psychotherapists in outpatient medical settings has also increased. In fact, we observe that outpatient cannabis-related diagnoses increased at a faster rate than the number of cannabis users, resulting in a higher proportion of users recognised with use problems. This result is difficult to interpret and there are at least three possible explanations. First, the recognition of problems linked to cannabis use has increased, resulting in a higher number of people seeking help for their problems. However, this would be a German-specific or European trend because opposite patterns were observed in the US with falling numbers of people seeking treatment for cannabis-related problems during periods of rising use prevalence [25, 26, 37]. It could be argued that the observed increased treatment rates are a consequence of increased police or court referrals, however, in Germany these referrals do not concern physicians or psychotherapists in outpatient settings and treatment rates increased substantially more than offences, so this explanation appears unlikely. Second, increased treatment uptake could be a result of more severe health problems per user. In Germany and most other European countries, cannabis potency has increased modestly for herbal and substantially for resin in the past decade [22]. Increasing potency levels in turn have been associated with greater health risks [31] and an observational study has linked variations in potency levels to treatment rates in the Netherlands [14]. Third, the trends merely reflect changes in the documentation, reflecting increased awareness of health care providers.

Interestingly, the largest increase in cannabis-related diagnoses was observed among older adults, i.e. those aged 35 and over. To some extent, this reflects a disproportionate increase in the prevalence of use in this age group. It is possible that the increasing average age of cannabis users reflects a longer history of use rather than increased initiation among older adults. This is supported by available estimates of age at first use, which have changed little over time (2015: 19.2 years; 2021: 19.4 years) and appear to be only slightly higher among older users (in 2021: 25–29 years: 18.4 years; 50–59 years: 23.8 years; [32, 35]). According to data from 20–30 years ago, less than 50% of German cannabis users aged 14–24 years stopped using cannabis within 10 years [30]. Given the available data, it is plausible to assume that a higher proportion of people today continue to use cannabis beyond the age of 30 or 40. There is very little evidence on the long-term effects of (intensive) cannabis use, but the risk of experiencing cannabis-related health problems appears to increase with accumulated exposure to cannabis [45], such as mild cognitive decline [24]. Conversely, older cannabis users may also adopt low-risk use strategies to avoid developing cannabis use problems [19].

Our findings suggest that cannabis use and related offences have risen less than outpatient cannabis-related diagnoses. For offences, we observe a stagnation in the last 2 years, perhaps owing to the COVID-19 pandemic. According to our findings, a sizeable, similar number of people using cannabis is affected by either health (3.7 per 1000 persons aged 15–64) or legal problems (3.1 per 1000 persons aged 12–59). These figures, however, capture only a fraction of persons with cannabis-related health problems (as not everyone with problems seeks help at all or help in the studied settings). Despite most offences may not be linked to severe legal or social consequences, the magnitude of potential legal problems linked to cannabis is not negligible: In Germany, the authorities may choose to not prosecute first-time violations [12], nonetheless, even the mere possession of minimal amounts of cannabis constitutes an offence against the narcotics law and can lead to a conviction (usually a fine). Moreover, those violations may also result in a suspension of the driving licence, with potential adverse implications for occupational and social life, while racial biases in cannabis arrests have been widely studied [17] and incarcerations are known to have detrimental health consequences (e.g. [18, 36]), respective data from Germany are not known to the authors. We can only add that the risk of legal consequences is highest for young male users.

Generally, the sequelae of law violations are still understudied and widely ignored from traditional health perspectives (see for example comprehensive and widely cited literature reviews of cannabis health risks: [6, 27]). Accordingly, public health indicators to evaluate cannabis policies do usually not consider the extent of legal problems or their (health) sequelae [13]. Given the extent of cannabis-related law offences in Germany, it appears prudent to consider them in evaluations of law changes [46].

## Conclusion

To the knowledge of the authors, this is the first study taking an interdisciplinary perspective in describing use, health, and legal time trends with regard to cannabis. We find rising figures for each indicator, suggesting a rise of consumption and related problems. Increasing treatment demand during times of rising consumption contrasts observations in the US and needs further investigation. Moreover, the health and social sequelae of law violations are poorly understood but should be considered for a comprehensive assessment of the cannabis burden for society.

## CRediT statement

Jakob Manthey: conceptualisation (lead); investigation (equal); methodology (lead); software (lead); validation (equal); formal analysis (lead); resources (lead); data curation (equal); writing—original draft (lead); writing—review and editing (lead); visualisation (lead); supervision (lead); project administration (lead); funding acquisition (lead). Moritz Rosenkranz: conceptualisation (support); investigation (support); validation (support); writing—review and editing (support); visualisation (support). Larissa Schwarzkopf: methodology (support); resources (support); writing—review and editing (support). Sinja Klinger: writing—review and editing (support).

## Supplementary Information

Below is the link to the electronic supplementary material.

Supplementary file1 (DOCX 854 KB)

## Data Availability

All data are publicly available in the figshare repository (https://www.doi.org/10.6084/m9.figshare.23850840).
