# Supplementary material for: Cannabis use, health problems, and criminal offences in Germany: national and state-level trends between 2009 and 2021
Source: Eur Arch Psychiatry Clin Neurosci. 2024 Mar 19;275(2):555–64. doi: 10.1007/s00406-024-01778-z (PMC11910392; doi:10.1007/s00406-024-01778-z)
Supplement: Supplementary file 1 — Supplementary file1 (DOCX 854 KB) [file 406_2024_1778_MOESM1_ESM.docx]

**SUPPLEMENTARY INFORMATION**

**Title:** Cannabis use, health problems, and criminal offences in Germany: national and state-level trends between 2009 and 2021

**Authors:**

Jakob Manthey, Sinja Klinger, Moritz Rosenkranz, Larissa Schwarzkopf

# Estimating 12-months cannabis use prevalence by state, sex, and age

To estimate the 12-months cannabis use prevalence by state, sex, and age, we relied on four data sources:

1. Prevalence estimates of the epidemiological survey of substance abuse (ESA; esa-survey.de or (1))
2. Prevalence estimates of the “Alkoholsurvey” (2)
3. Hospital admissions for F12 (3)
4. Population data from the federal statistical office (4)

The ESA served as the main input source as it provides national estimates at every wave (2009, 2012, 2015, 2018, 2021), in addition to state-level estimates for select states and waves. The availability of state-level data is summarized in ***Supplemental Table 1***.

***Supplemental Table 1*.** Data availability of 12-months cannabis use prevalence

| **State** | **Years with available data** |
| --- | --- |
| Bayern | 2009;2015;2021 |
| Berlin | 2012;2018;2021 |
| Bremen | 2021 |
| Hamburg | 2009;2015;2021 |
| Hessen | 2015;2018 |
| Nordrhein-Westfalen | 2015;2018;2021 |
| Sachsen | 2009;2015;2018;2021 |
| Sachsen-Anhalt | 2021 |
| Thüringen | 2012;2015;2018 |

In most cases, the state-level estimates were not available by sex and age. Of n=143 estimates, n=16 estimates concerned 18-64-year-olds (n=8 for men, n=8 for women), while the remaining n=127 estimates concerned various, partially overlapping age groups (18-24, 25-29, 25-39, 30-39, 40-49, 50-64, 40-59, 60-64, 18-64). Given the structure of the data available, we estimated the prevalence in two steps:

1. Age-specific (but not by age) prevalence for all states between 2009 und 2021
2. Age- and sex-specific prevalence for all states between 2009 und 2021

For 1.) age-specific (but not by age) prevalence for all states between 2009 und 2021, we assumed that the geographical variation in use prevalence is proportional to the geographical variation in hospitalisation rate for F12 diagnoses. The absolute and relative difference between the national and federal indicators is shown in Supplementary Figure 2 for those states with data available. It confirms the plausibility of our assumption, especially for younger adults (18-24 years; pearson correlation between difference of prevalence and hospitalisation rate for relative and absolute indicator: r=0.544 and r=.385). On this basis, we predicted the ratio of state-level to federal prevalence using the ratio of state-level to federal hospitalisation rate, adjusting for age and year effects. The best model was determined iteratively and included interaction terms of hospitalisation rate and age as well as hospitalisation rate and year (R² = 31,1%; F-statistic: 4.869) and the model equation is given in Equation 1:

Equation 1:

$$y=\beta_{0}+ \beta_{1}*x_{1}+\beta_{2}*x_{2}+\beta_{3}*x_{3}+\beta_{4}*x_{1}*x_{2}+\beta_{5}*x_{1}*x_{3}+\varepsilon$$

- $y$ = Ratio of state-level use prevalence to federal use prevalence
- $x_{1}$ = Ratio of state-level hospitalisation rate to federal hospitalisation rate
- $x_{2}$ = Dummy variable for age group 25-39
- $x_{3}$ = Dummy variable for age group 40-59
- $\beta_{0}$ = Intercept (mean for age group 18-24)
- $\beta_{1}$ = Regression coefficient for $x_{1}$
- $\beta_{2}$ = Regression coefficient for $x_{2}$
- $\beta_{3}$ = Regression coefficient for $x_{3}$
- $\beta_{4}$ = Regression coefficient for interaction of $x_{1}$ and $x_{2}$
- $\beta_{5}$ = Regression coefficient for interaction of $x_{1}$ and $x_{3}$
- $\varepsilon$ = random error

As shown in ***Supplementary Figure 2***, cannabis use prevalence was adequately modelled for the states with available data. Accordingly, we used that model to impute the missing values for the other states.

Next, we obtained consistent time series for the 13 years, based on the estimates for the years 2009, 2012, 2015, 2018, and 2021. We distinguished between states a) without any or at most one empirical prevalence estimates (Baden-Württemberg, Brandenburg, Bremen, Mecklenburg-Western Pomerania, Lower Saxony, Rhineland-Palatinate, Saarland, Saxony-Anhalt, Schleswig-Holstein) and b) with at least two empirical prevalence estimates (Bavaria, Berlin, Hamburg, Hesse, North Rhine-Westphalia, Saxony, Thuringia). For the first group and within each state, we conducted LOESS regressions with the five data points and year was the sole covariate. Based on this model, prevalence estimates were predicted for the remaining years. For the second group and within each state, we pursued a 2-step solution. First, we conducted a linear regression while the empirical prevalence estimates were assigned greater weights than the statistically derived prevalence estimates (ratio 2:1). Using the predicted values from this model for all years with missing data, we obtained a complete time series for each state. Second, we performed a LOESS regression to obtain smoothed estimates for these states, as illustrated in ***Supplementary Figure 3***.

Now we had a complete time series of prevalence estimates for every state, for the age groups 18-24, 25-39, and 40-59. To also capture cannabis use among minors, we used the ratio of 18-24-year-olds to 12-17-year-olds from the second population-based survey. That survey does not disclose estimates by federal state, but it does reveal estimates for the years 2008, 2010, 2011, 2012, 2014, 2015, 2016, 2018, 2019, and 2021. In the overlapping age group of 18-24-year-olds, a very similar, increasing trend in cannabis use between both surveys can be observed. With a linear regression, the ratio of cannabis use prevalence among 18-24-year-olds to 12-17-year-olds was averaged for all years and applied to the data to obtain prevalence estimates for every state and year for 12-17-year-olds.

For 2.) the age- and sex-specific prevalence for all states between 2009 und 2021, we combined the consistent time series of prevalence estimates by state and age group with the sex distribution. As shown in Supplementary Figure 4, the gender ratio shrinked over time but did not differ systematically between age group 18-64 (ESA) or age groups 12-17 and 18-25 (Alkoholsurvey). Accordingly, we estimated the ratio between women and total as well as men and total as a function of time with a linear regression: between 2009 and 2021, the ratio in use prevalence between women and total increased from 0.66 to 0.78 and decreased between men and total from 1.32 to 1.21. Applying these ratios, we estimated the 12-months cannabis use prevalence for all states, by age, sex and year.

Time trends of the final estimates were compared to the empirical estimates, by sex and age for the federal level. As shown in Supplementary Figure 4, very similar trends can be observed, while our estimates are slightly above those from the two surveys. In 2021, the difference between our cannabis use prevalence and the ESA estimates was 1 to 1.5 percentage points (18-24 years: 24.2% vs 22.8%; 25-39 years: 13.4% vs. 12.3%; 40-59 Jahre: 5.7% vs. 4.6%), which fall within the reported 95% confidence interval (e.g., prevalece for 18-64-year-olds in 2021: 8.8%, 95% CI: 7,7 – 10,0%; (1)).

For external validation, we further compared our estimates with empirical estimates from two recently conducted surveys. In a national survey from 2022, use prevalence among 25-39-year-olds was estimated at 8.2% (95% CI: 7.1-9.5%; unpublished data). We find considerably higher estimates for the same age group for the year 2021 (13.4%). The other survey was conducted in Berlin in 2022 among young adults. Among 18-24-year-olds, 12-months use prevalence was estimated at 28.5% (men: 34.7%; women: 22.4%; own calculations, based on (5)). We find slightly lower estimates for the same age group for the year 2021 (men: 31.0%; women: 20.0%, total: 25.5%).

Overall, it can be concluded that we have produced prevalence estimates that are comparable with estimates from available surveys. Differences in estimates may be driven by sampling differences as well as a considerable degree of underreporting (e.g., nearly 50% as estimated for Sweden: (6)).

# Supplementary Figures


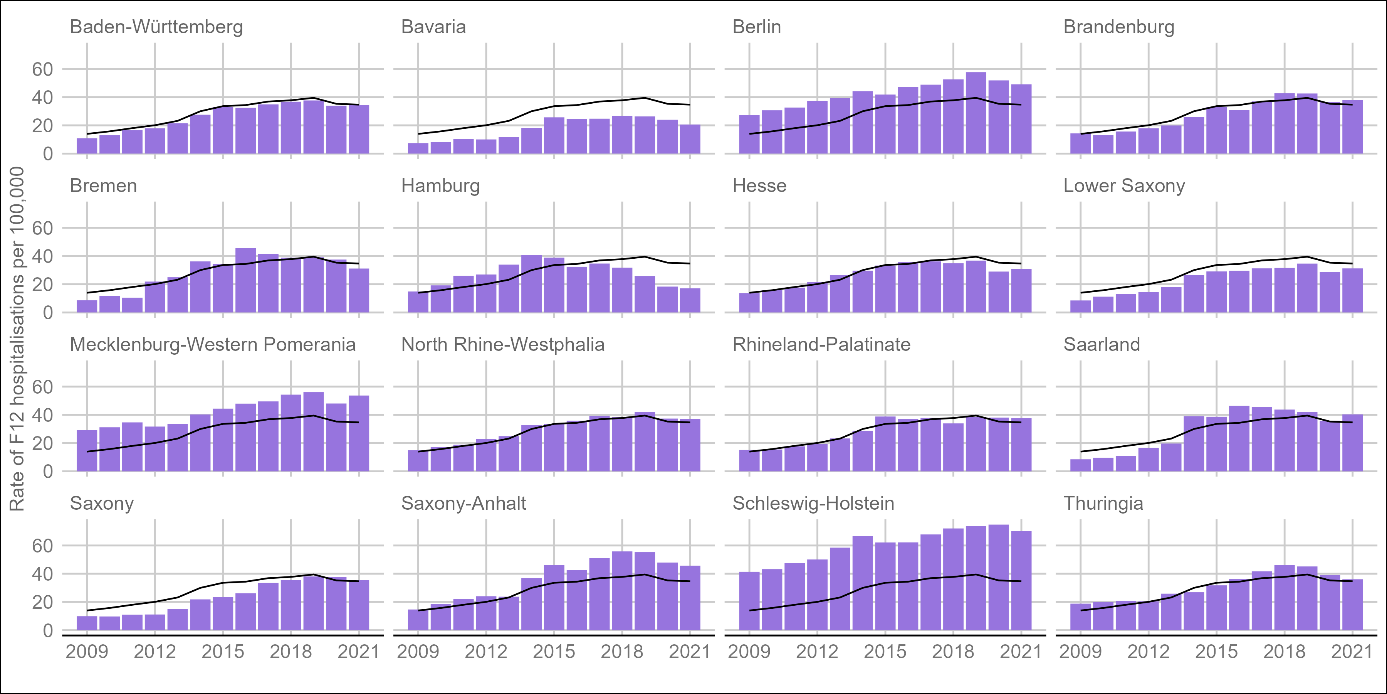


**Supplementary Figure 1.** Rate of hospitalisations per 100,000 (not age-standardized) by federal state between 2009 and 2021. The black line illustrates the nationwide average. The rates are calculated for the population aged 12-59, assuming that nearly all observed hosptitalisations fall into this age bracket.


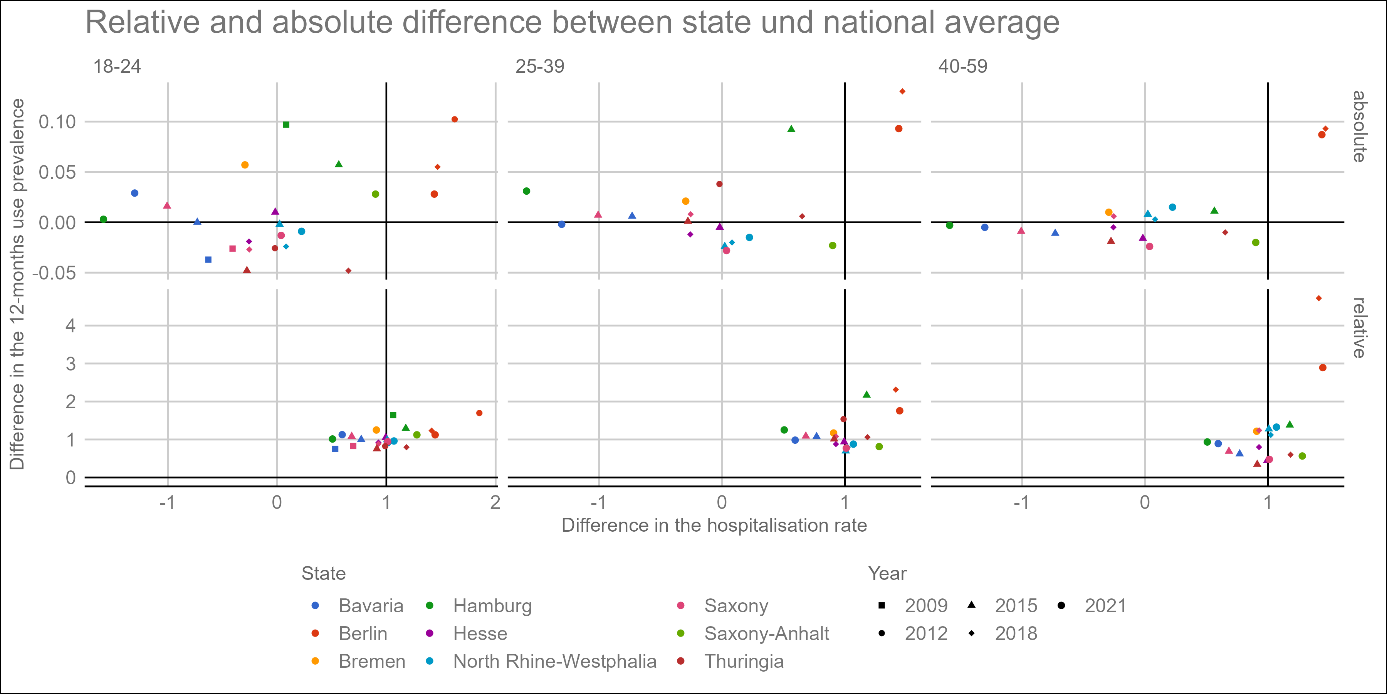


**Supplementary Figure 2.** Absolute (top) and relative (bottom) difference between state-level and national hospitalisation rate (x-axis) as well as between state-level and national 12-month prevalence of consumption (y-axis), by state (color), year (shape), and age group (columns)


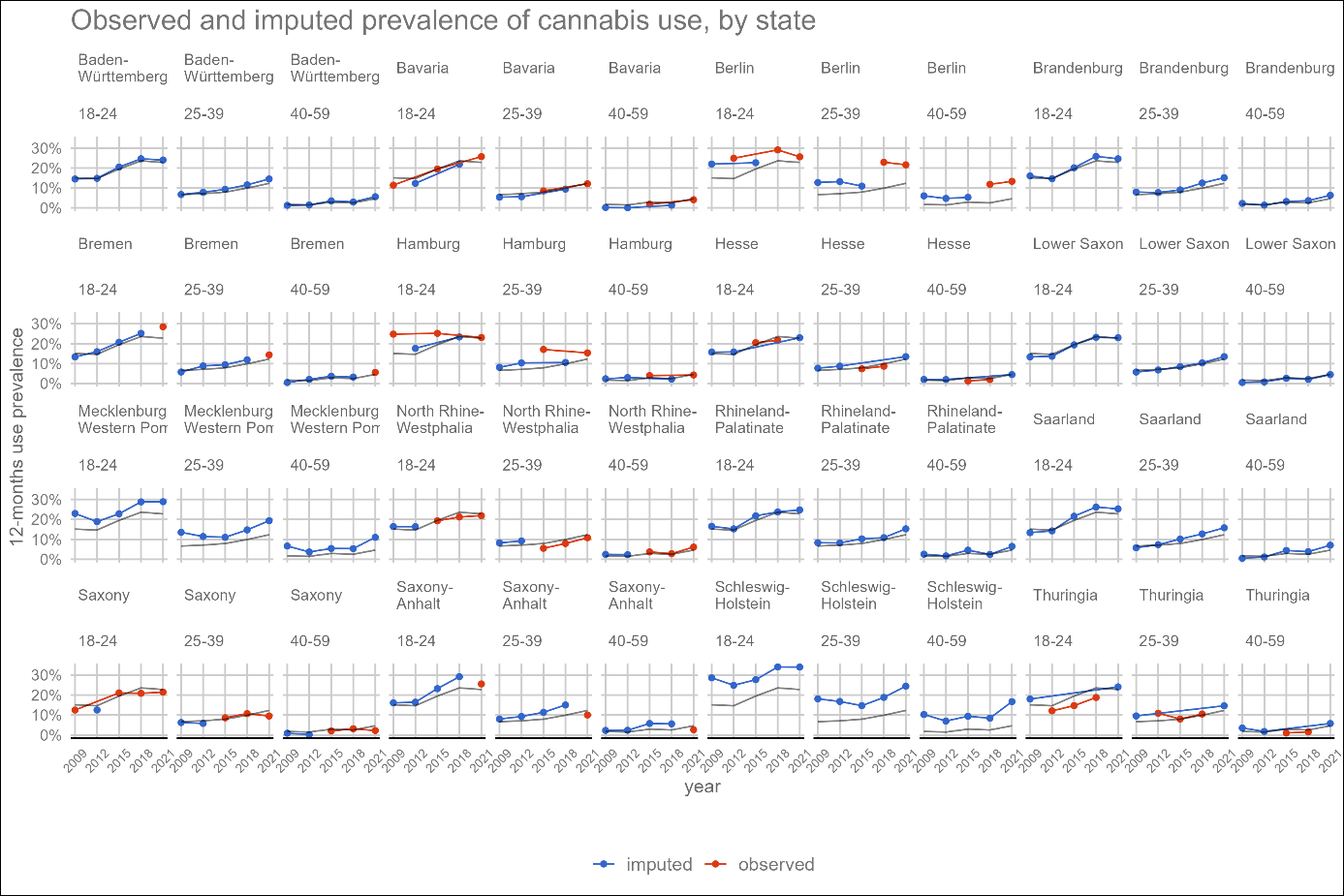


**Supplementary Figure 3.** Observed (red) and imputed (blue) 12-months-prevalence of cannabis consumption by state and age. The gray line represents the nationwide average in each age-group.


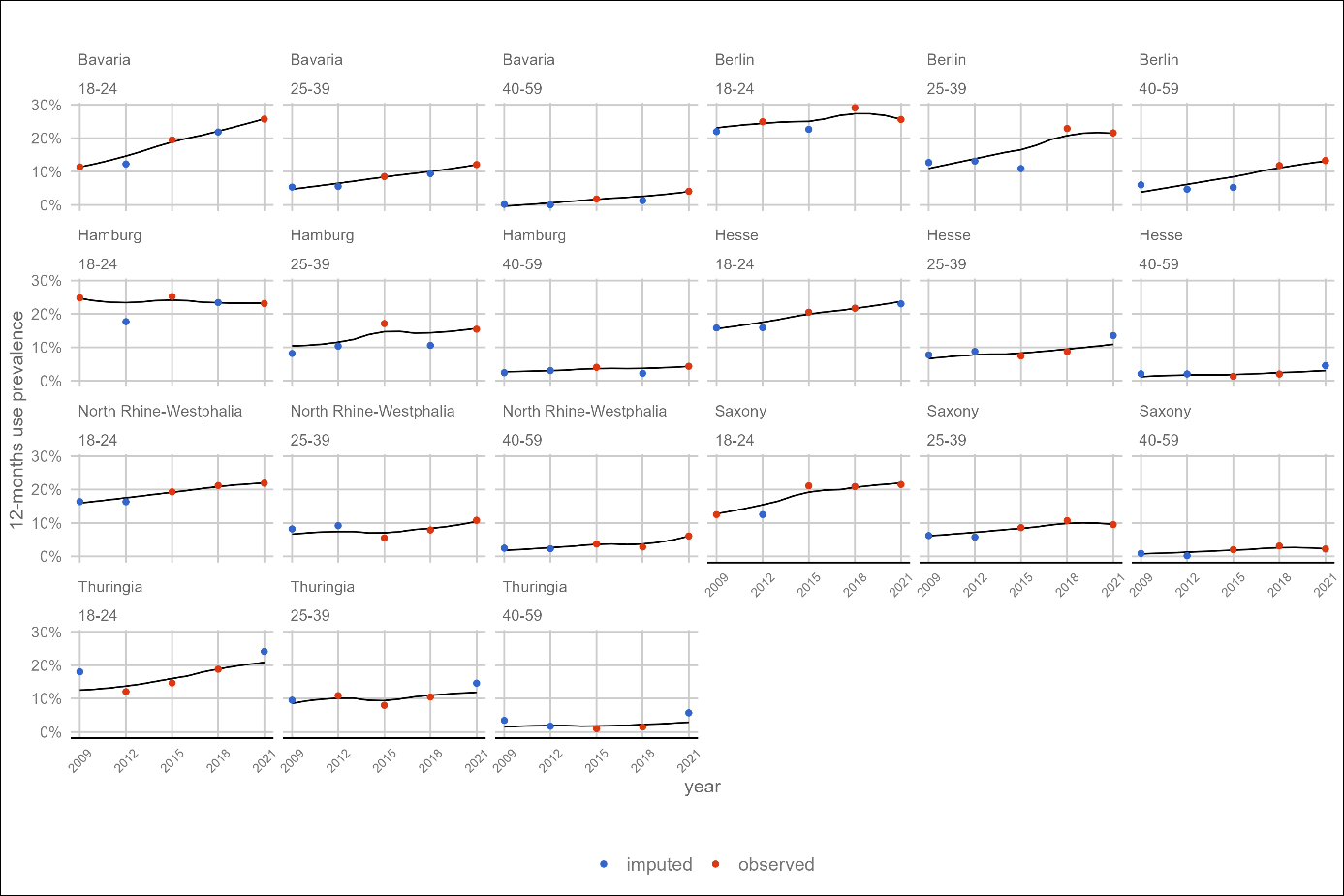


***Supplementary Figure 4.*** *12-months prevalence of cannabis consumption in german federal states by age group. The dots represent observed (red) and imputed (blue) data. The black line shows the time trend, which was estimated with a 2-step process (linear regression and LOESS).*


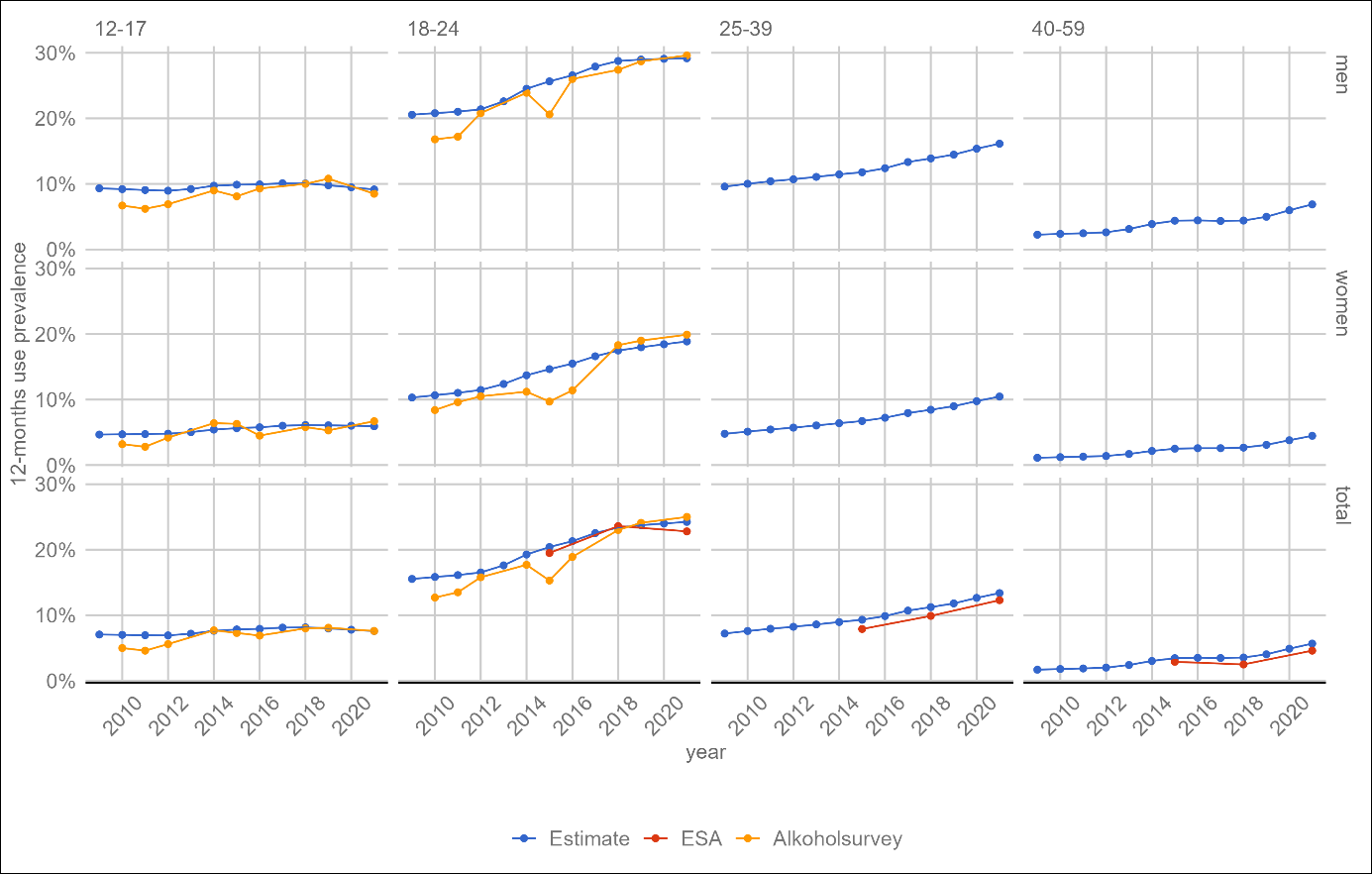


**Supplementary Figure 5.** Estimation of the 12-month prevalence of consumption in Germany by age and sex and compared to data from two surveys: ESA and ‘Alkoholsurvey’.


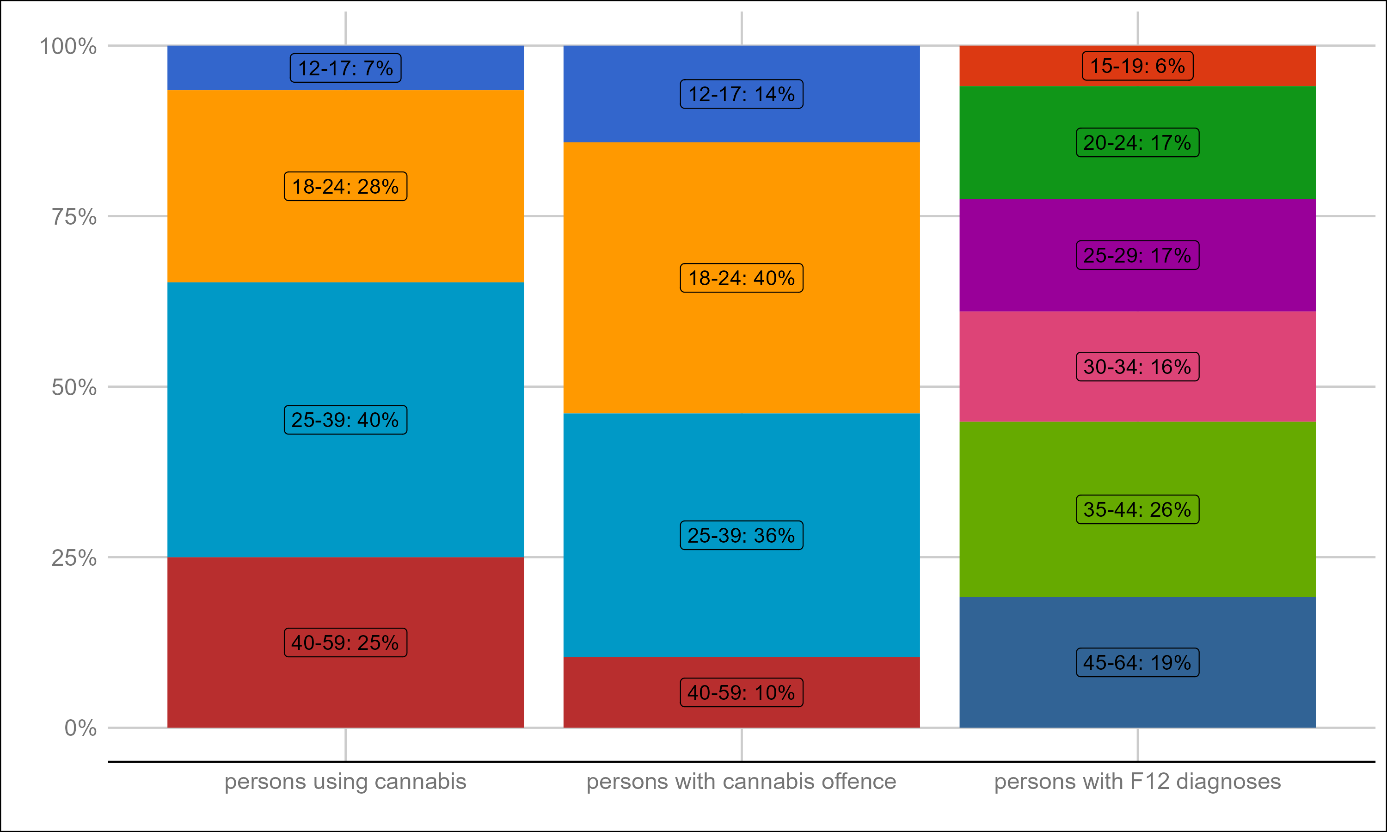


**Supplementary Figure 6.** A breakup of the absolute number of a) persons using cannabis, b) persons with registered offences for possessing cannabis, and c) persons with F12 diagnoses in 2021, by age group.


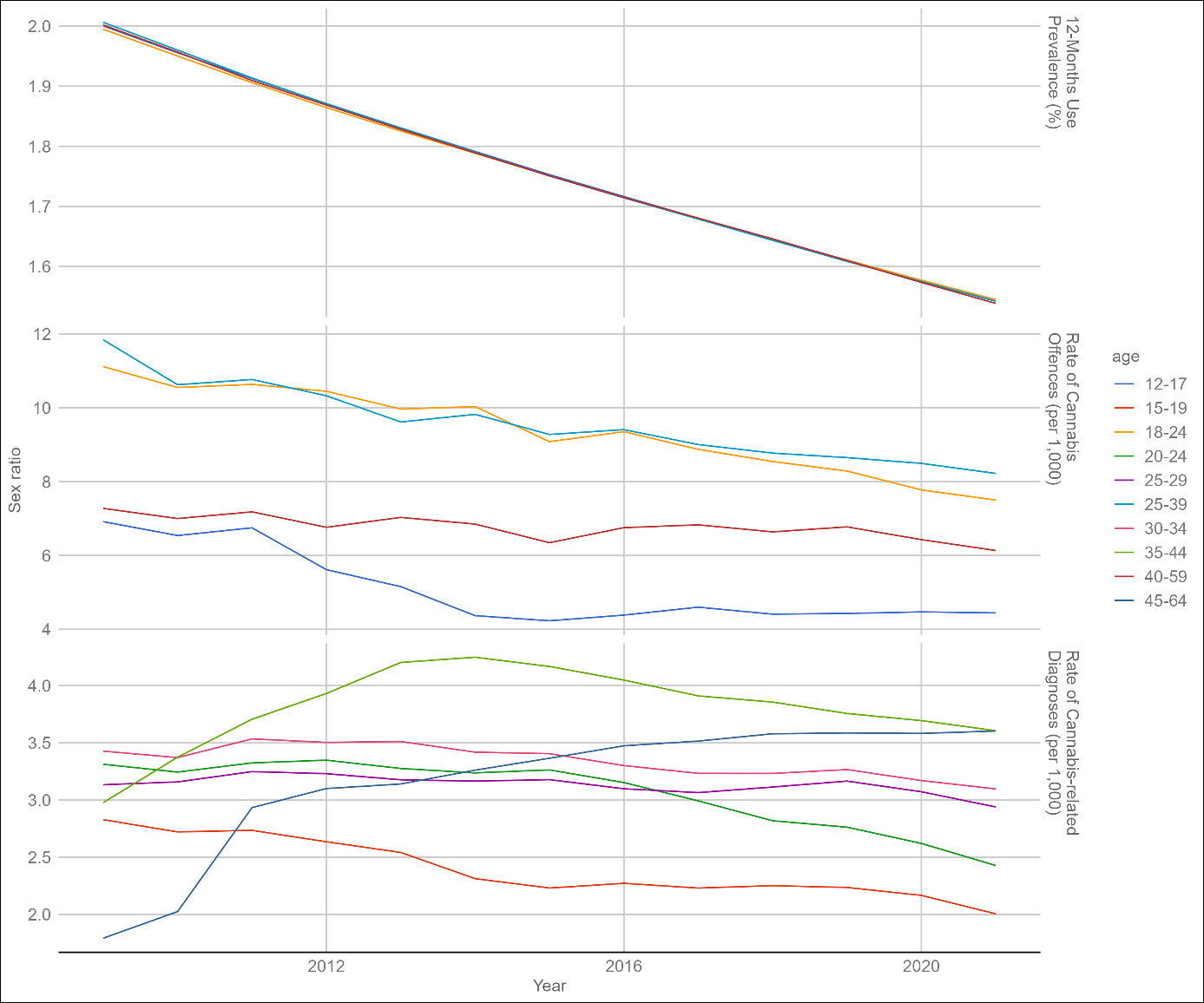


**Supplementary Figure 7.** Time trend of sex ratio (=men / women) over time for the three indicators (cannabis use prevalence, rate of cannabis offences, rate of cannabis-related diagnoses) and by age group.

# References

1. Rauschert C, Möckl J, Seitz NN, Wilms N, Olderbak S, Kraus L. The Use of Psychoactive Substances in Germany-Findings From the Epidemiological Survey of Substance Abuse 2021. Deutsches Arzteblatt international. 2022;119(31-32):527-34.

2. Orth B, Merkel C. Der Substanzkonsum Jugendlicher und junger Erwachsener in Deutschland. Ergebnisse des Alkoholsurveys 2021 zu Alkohol, Rauchen, Cannabis und Trends. Köln: Bundeszentrale für gesundheitliche Aufklärung; 2022.

3. Gesundheitsberichterstattung des Bundes. Diagnosedaten der Krankenhäuser ab 2000 (Eckdaten der vollstationären Patienten und Patientinnen). Gliederungsmerkmale: Jahre, Behandlungs-/Wohnort, ICD10. Bonn: Statistisches Bundesamt; 2023.

4. Destatis. 12411-0013: Bevölkerung: Bundesländer, Stichtag, Geschlecht, Altersjahre Wiesbaden: Statistisches Bundesamt; 2020 [Available from: <https://www-genesis.destatis.de/genesis/online>.

5. Kalke J, Rosenkranz M. Cannabiskonsum von Jugendlichen und jungen Erwachsenen in Berlin: Ergebnisse einer Bevölkerungsumfrage2023 21 June 2023. Available from: <https://www.isd-hamburg.de/wp-content/uploads/2023/06/Bericht_CannabisBerlin.pdf>.

6. Andersson F, Sundin E, Magnusson C, Ramstedt M, Galanti MR. Prevalence of cannabis use among young adults in Sweden comparing randomized response technique with a traditional survey. Addiction. 2023.
